# How Ought We to Dispose of the Dead?

**Published:** 1888-03

**Authors:** 


### HOW OUGHT WE TO DISPOSE OF THE DEAD.

Sir Spencer Wells, the famous English Surgeon in the course of a recent address before a Nottingham Medical Society, upon sanitary progress and cremation, said : " There is one of our customs on the injurious influence of which upon the public health even the medical profession has thought too little. It is true that ever since the dawn of the sanitary age the most common mode of disposal of the dead by burial in the earth has been denounced as dangerous, and much has been done to stop burial within churches and in graveyards in large towns. Suburban cemeteries have replaced in many districts the town churchyard. But, as population increases, it has become evident that more than this must be done if the living are to be protected from the dead. Many of our suburban cemeteries are now as dangerous as town graveyards were fifty years ago.

Signs are not wanting that public sentiment is revolting against the present modern slow process of putrefactive corruption, and accepting the purer and more rapid ancient process of purifying cremation. We want the help of the whole profession in making the dangers of earth burial and the advantages of cremation more widely known. And we want the family advisers of the people in all sanitary matters, especially to consider and to insist upon the absolute necessity of this sanitary reform if we are ever to succeed in stamping out infective diseases.

Seven years ago at Cambridge I alluded to Darwin's paper on the formation of mould, published in 1837, to show how in old pasture land every particle of the superficial layer of earth overlying different kinds of subsoil, has passed through the intestines of earth worms. And I described the researches of Pasteur on charbon, or splenic fever in cattle, proving that the superficial mould in a field where diseased cattle have been buried may contain the specific germs which propagate the disease, and that the same specific germs are found within the intestines of the earth worms—germs which, inoculated, propagate the disease. What has been proved to be true of charbon may be true of small-pox, typhoid, scarlet fever, or measles, of yellow fever, malarial fever, cholera, and typhoid. Scarlet fever is just now in London a question of greater urgency ; although Dr. Ogle informs me that, notwithstanding the popular alarm, the deaths have in reality, " been considerably below the average for the previous forty-seven years." Admitting this, still the deaths in the thirteen weeks from July 23 to Oct. 15 in the metropolitan district have risen weekly from 22 to 56, the total deaths in the quarter having amounted to 476.

Every one of these dead bodies contained myriads of the specific organism, or the germs upon which the fever depends, and they are all now putrefying in the earth some five or ten feet below the surface of the soil round London. The germs or spores may retain their latent vitality for many years, like a grain of corn or a flower seed, and may be ready to germinate and communicate the specific disease when favorable conditions arise. Mr. Wheelhouse of Leeds informs me that he has known scarlet fever so spread after thirty years burial. He writes to me that he once witnessed an outbreak of scarlet fever in the family of a clergyman, which, in my opinion was unquestionably caused by the removal of some soil from a portion of the graveyard which, thirty years previously, had been used as the burial place for those who had died of an epidemic of scarlet fever. Renovations of the churchyard left some of the soil unused: and, rather than permit it to be thrown heedlessly away, it was taken into the clergyman's garden, which adjoined, and spread out over some beds around his nursery. Only a few days after, scarlet fever broke out among his children in a virulent form.

Can any one doubt that if this was observed in one place, it must have occurred in others where it has escaped notice, or that the 476 bodies of scarlet fever patients buried during the past three months around London (some 50,000 pounds by weight of animal matter slowly undergoing putrefactive decomposition in the earth, charged with myriads of parasitic organisms, the germs or spores of which may retain their vitality for many years) may be storehouses of poison preserved for the destruction of future generations of the coming race?

If you acknowledge the importance of the question, and advocate cremation as the best mode hitherto practiced, you may be called upon to answer various objections—legal, medico-legal, religious, sentimental. The legal need not trouble you; the highest legal authorities, have decided that cremation is not illegal. The medico-legal objection, that a murdered or poisoned body might be burned, and that discovery of crime by subsequent exhumations would be then impossible, is easily answered by the argument that under proper regulations no human body could possibly be burned, without far more positive evidence of death from natural or known causes than is now required before burial, and that by promoting cremation you are also leading to a more correct and general system of registration and certificates of the true causes of death. The religious objection is met by the words of the late Bishop of Manchester and of Canon Liddon. A good many years ago Wordsworth, then Bishop of Lincoln, was supposed to have made some such objection to cremation. He did not really do so, but he was supposed to have so objected, and the late Lord Shaftesbury about this time said to me: "What an audacious limitation of the power of the Almighty! What has become of the blessed martyrs who were burned at the stake?" And writing to me in April, 1885—almost the last letter he ever wrote—he, used almost the same words. After this, I hardly think religious feeling will do much to retard the change from burial to cremation. And I have found the feeling of ministers of religion, to be influenced by the consideration, that the association of the family with their place of worship is better preserved by cremation than by burial. The harmless ashes of the departed of a large population may be preserved in urns within our churches, beneath memorial windows or brasses, or in cloisters close to the church, even in the midst of large towns, instead of the bodies being sent to distant cemeteries and the association of church and family thus weakened.

Cremation has been spoken of as an expensive process. There cannot be a greater mistake. It is very much cheaper than burial in the earth. There need be no coffin—a clear saving—and no grave has to be purchased. Scarcely any land is wanted, even for the crematorium of a large city or district. Burial in London alone has required twenty-two cemeteries, which occupy more than 2,200 acres of land. In England and Wales there are more than 11,000 cemeteries, and the necessity for new cemeteries is continually arising. In 1851 a report to the House of Commons showed that the average cost per acre of land purchased for cemeteries was £123. Probably the cost of land near London was much more, but at this estimate the cost of the land alone of the metropolitan cemeteries has been more than a quarter of a million of money, the annual interest at 3 per cent amounting to nearly £8,000. A very large part of this would be saved as cremation became more general. Then the other funeral expenses would be smaller. In 1884 it was estimated that nearly £5,000,000 was expended in one year in funerals. The Cremation Society for a payment of ten guineas undertakes now to perform cremation. It could be easily proved that if the practice became general the cost to a parish or union, including removal of a body to a neighboring crematory, need not exceed ten shillings for each cremation.

The strongest objection which the advocates of cremation meet is the one which is termed sentimental. The fear of offending the feelings of relatives and dear friends, the disinclination at a time of bereavement and suffering to depart from ordinary routine or custom, lead many, as

Dr. Cameron says, to lack the energy and decision to carry their convictions into practice. I firmly believe from my own experience that, however painful it may be to make people understand what a revolting change takes place after burial in the earth in the bodies of those they have loved, for how long a time it goes on and how dangerous it is to the living, very little more is required to call forth a purer sentiment in favor of rapid purification by fire.

